# Supplementary material for: An alternative splicing modulator decreases mutant HTT and improves the molecular fingerprint in Huntington’s disease patient neurons
Source: Nat Commun. 2022 Nov 10;13:6797. doi: 10.1038/s41467-022-34419-x (PMC9649613; doi:10.1038/s41467-022-34419-x)
Supplement: Supplementary file 1 — Supplementary Information [file 41467_2022_34419_MOESM1_ESM.pdf]

An alternative splicing modulator decreases mutant HTT and improves the molecular fingerprint in Huntington's disease patient neurons

Florian Krach[1], Judith Stemick[2], Tom Boerster[1], Alexander Weiss[3], Ioannis Lingos[3], Stephanie Reischl[1], Holger Meixner[2], Sonja Ploetz[2], Michaela Farell[1], Ute Hehr[4], Zacharias Kohl[5], Beate Winner[1,6*§], Juergen Winkler[2, 6*§]

[1] Department of Stem Cell Biology, University Hospital Erlangen, Friedrich-Alexander University of Erlangen-Nürnberg (FAU), Germany

[2] Department of Molecular Neurology, University Hospital Erlangen, Friedrich-Alexander University of Erlangen-Nürnberg (FAU), Germany

[3] Evotec SE, Hamburg, Germany.

[4] Zentrum für Humangenetik Regensburg, Regensburg, Germany

[5] Department of Neurology, University of Regensburg, Germany

[6] Center of Rare Diseases Erlangen (ZSEER), University Hospital Erlangen, Friedrich-Alexander University of Erlangen-Nürnberg (FAU), Germany

*Correspondence: juergen.winkler@uk-erlangen.de and beate.winner@fau.de

[§] Jürgen Winkler and Beate Winner contributed equally to this work.

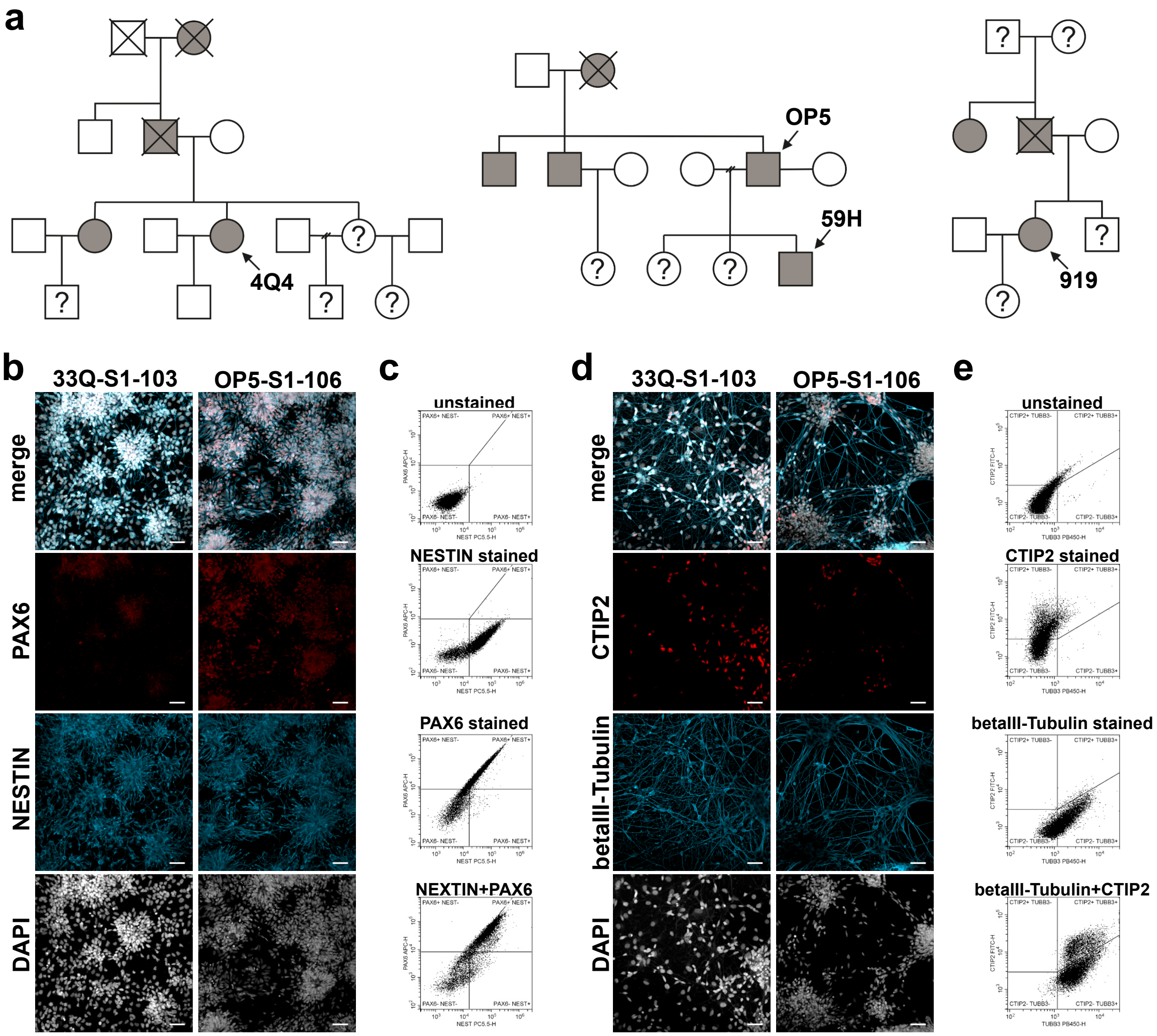

Supplementary Figure 1 Differentiation of iPSC into cortical neurons

a Pedigree of the patient cohort in this study

b representative pictures of cortical progenitors. Scale bar: 50µm.

c gating strategy for FACS analysis of cortical neurons.

d representative pictures of cortical progenitors. Scale bar: 50µm.

e gating strategy for FACS analysis of cortical neurons.

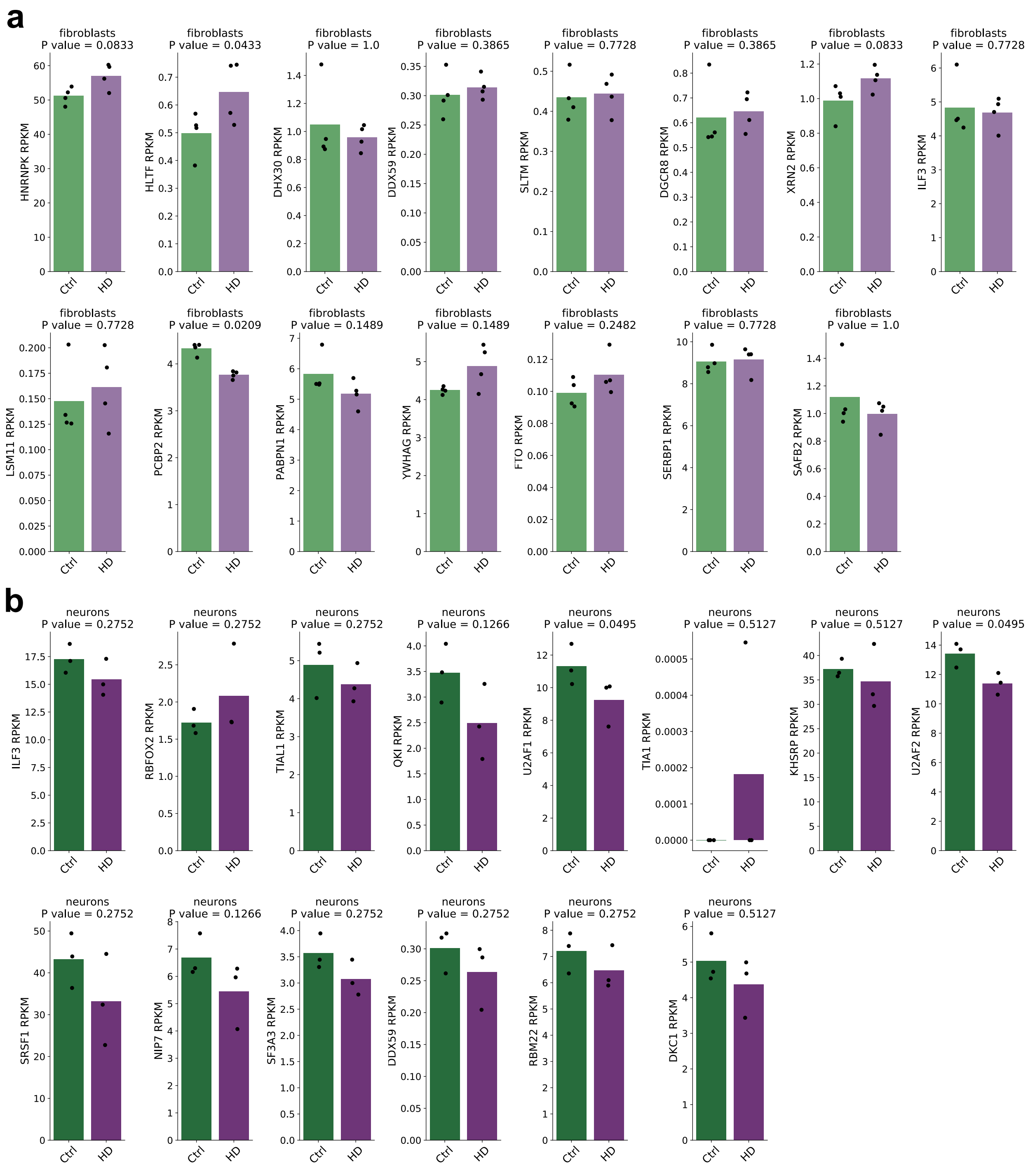

Supplementary Figure 2 Gene expression in fibroblasts and cortical neurons of RBP targets identified via eCLIP-seq

a RPKM values in Ctrl (green) and HD (purple) fibroblasts of RBPs where binding was found to be enriched at AS events changed in HD fibroblasts. Statistics: Mann-Whitney test. P value depicted on top of each graph. Bars: median. Individual values as dots.

b RPKM values in Ctrl (green) and HD (purple) cortical neurons of RBPs where binding was found to be enriched at AS events changed in HD cortical neurons. Statistics: Mann-Whitney test. P value depicted on top of each graph. Bars: median. Individual values as dots.

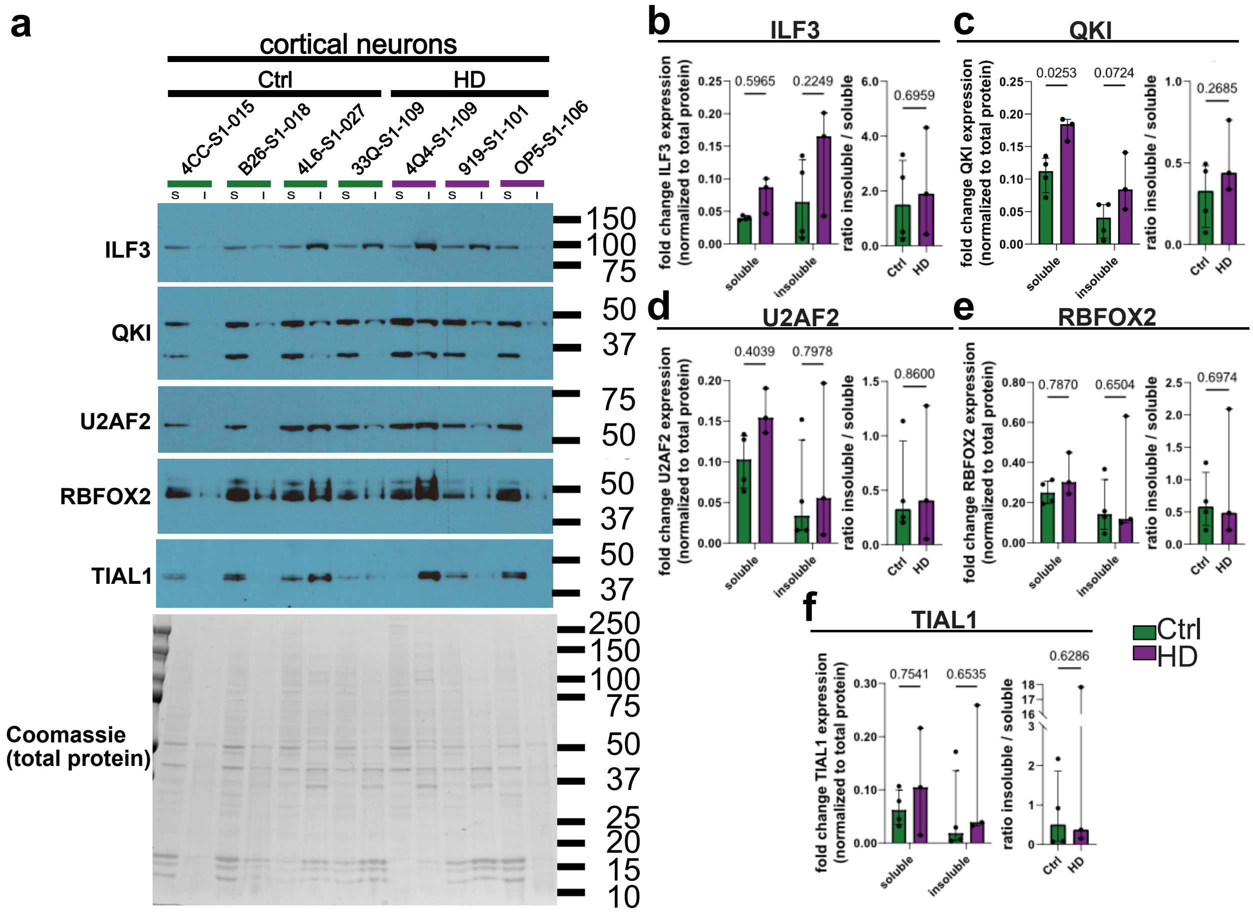

Supplementary Figure 3 Investigation of candidate RBP solubility in cortical neurons

a Western blot images of RIPA soluble (S) and RIPA insoluble (I) fractions of 5 candidate RBPs (ILF3, QKI, U2AF2, RBFOX2, and TIAL1) where enrichment of binding was determined in HD cortical neuron AS events (Figure 2H). Coomassie stained gel was stained to illustrate total protein in the fractions. Solubility fractionation was performed in n=4 Ctrl and n=3 HD cortical neuron lines. Source data are provided as a Source Data file.

b-f quantification of absolute soluble and insoluble signal (left graph) and the ratio of insoluble/soluble signal (right graph) of signal normalized to Coomassie in Ctrl (green bars) and HD (purple bars) of ILF1 (**b**), QKI (**c**), U2AF2 (**d**), RBFOX2 (**e**) and TIAL1 (**f**). ILF1 statistics: absolute values (left graph): 2-way ANOVA: solubility P = 0.2288; disease P = 0.0836; interaction P = 0.6535. P values on top of graph depict significance of Šídák's multiple comparison test. Ratios (right graph): Welch's test (P value on top of graph). QKI statistics: absolute values (left graph): 2-way ANOVA: solubility P = 0.0003; disease P = 0.0319; interaction P = 0.4628. P values on top of graph depict significance of Šídák's multiple comparison test. Ratios (right graph):

Welch’s test (P value on top of graph). U2AF2 statistics: absolute values (left graph): 2-way ANOVA: solubility P = 0.1106; disease P = 0.2698; interaction P = 0.6258. P values on top of graph depict significance of Šídák’s multiple comparison test. Ratios (right graph): Welch’s test (P value on top of graph). RBFOX2 statistics: absolute values (left graph): 2-way ANOVA: solubility P = 0.5162; disease P = 0.3331; interaction P = 0.8808. P values on top of graph depict significance of Šídák’s multiple comparison test. Ratios (right graph): Welch’s test (P value on top of graph). TIAL1 statistics: absolute values (left graph): 2-way ANOVA: solubility P = 0.9084; disease P = 0.1671; interaction P = 0.9290. P values on top of graph depict significance of Šídák’s multiple comparison test. Ratios (right graph): Mann-Whitney test (P value on top of graph). Bars: median ± IQR. Source data are provided as a Source Data file.

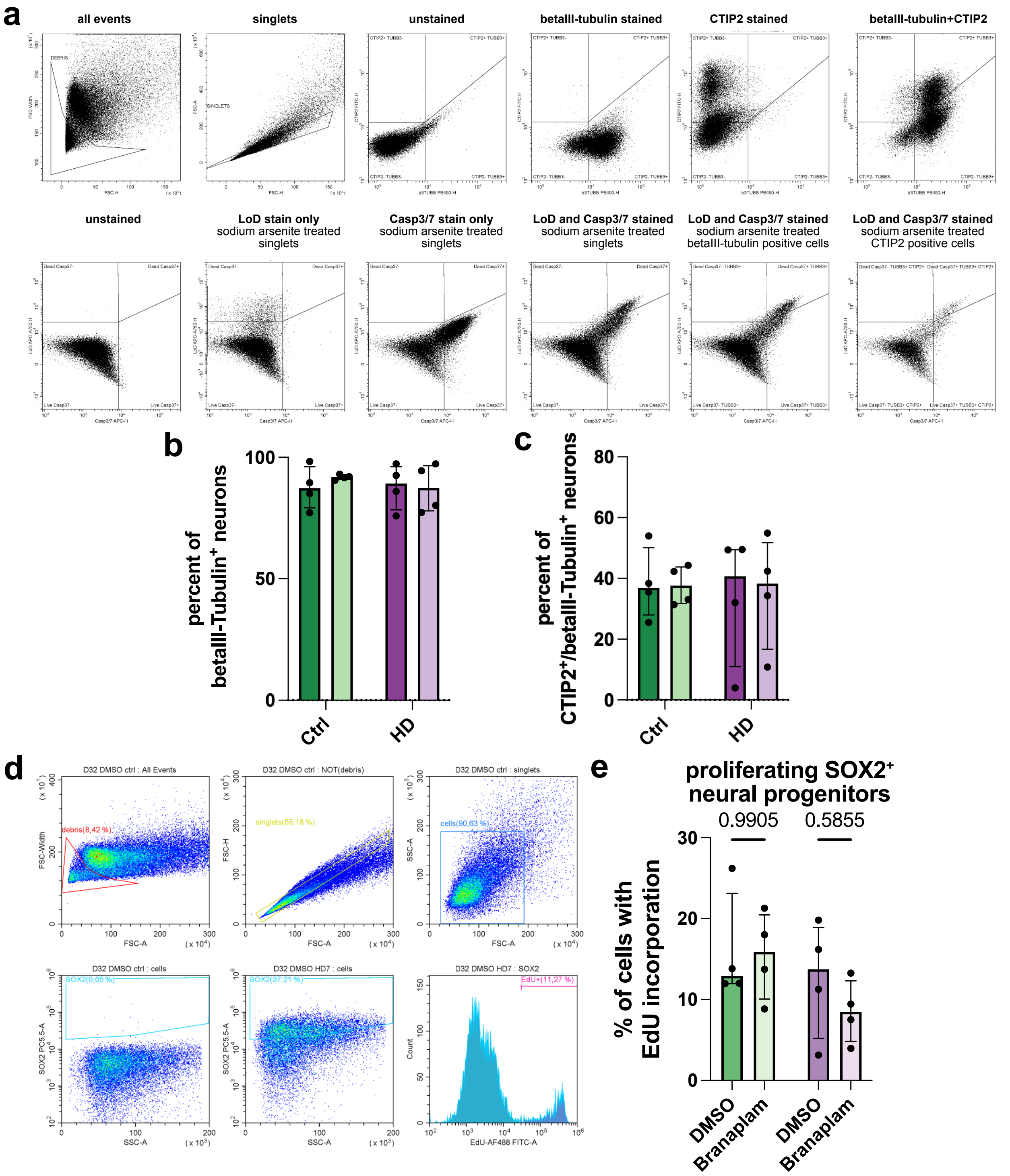

Supplementary Figure 4 FACS gating analyses of apoptosis cells and proliferating cortical progenitor cells

a gating strategy for FACS analysis of cortical neurons.

b Bar plot representing quantification of betaIII-Tubulin positive cells at day 50 of differentiation. Statistics: 2-way ANOVA (DMSO vs. Branaplam: P = 0.6164; Ctrl vs. HD: P = 0.6674; interaction: P = 0.5057). Bars: median ± IQR.

c Bar plot representing quantification of CTIP2 / betaIII-Tubulin double-positive cells at day 50 of differentiation. Statistics: 2-way ANOVA (DMSO vs. Branaplam: P = 0.8756; Ctrl vs. HD: P = 0.7551; interaction: P = 0.7692). Bars: median ± IQR.

d FACS gating strategy of cortical progenitor cells stained with labeled with EdU. Debris is gated out first (top left), singlets are selected (top middle) and cells to be analyzed selected (top right). SOX2-positive neural progenitor cells are gated out (bottom left: unstained and middle: stained), and neural progenitors with incorporated EdU are quantified (bottom right).

e Bar graph depicting percent of $SOX2^+$ neural progenitor cells with EdU incorporation in control (n=4, green bars) and HD (n=4, purple bars) cells treated with DMSO or 10µM Branaplam for 3 days. Statistics: 2-way ANOVA (DMSO vs. Branaplam: P = 0.1428; Ctrl vs. HD: P = 0.4619; interaction: P = 0.5851). P values on top of graph depict significance of Šídák's multiple comparison test. Bars: median ± IQR.

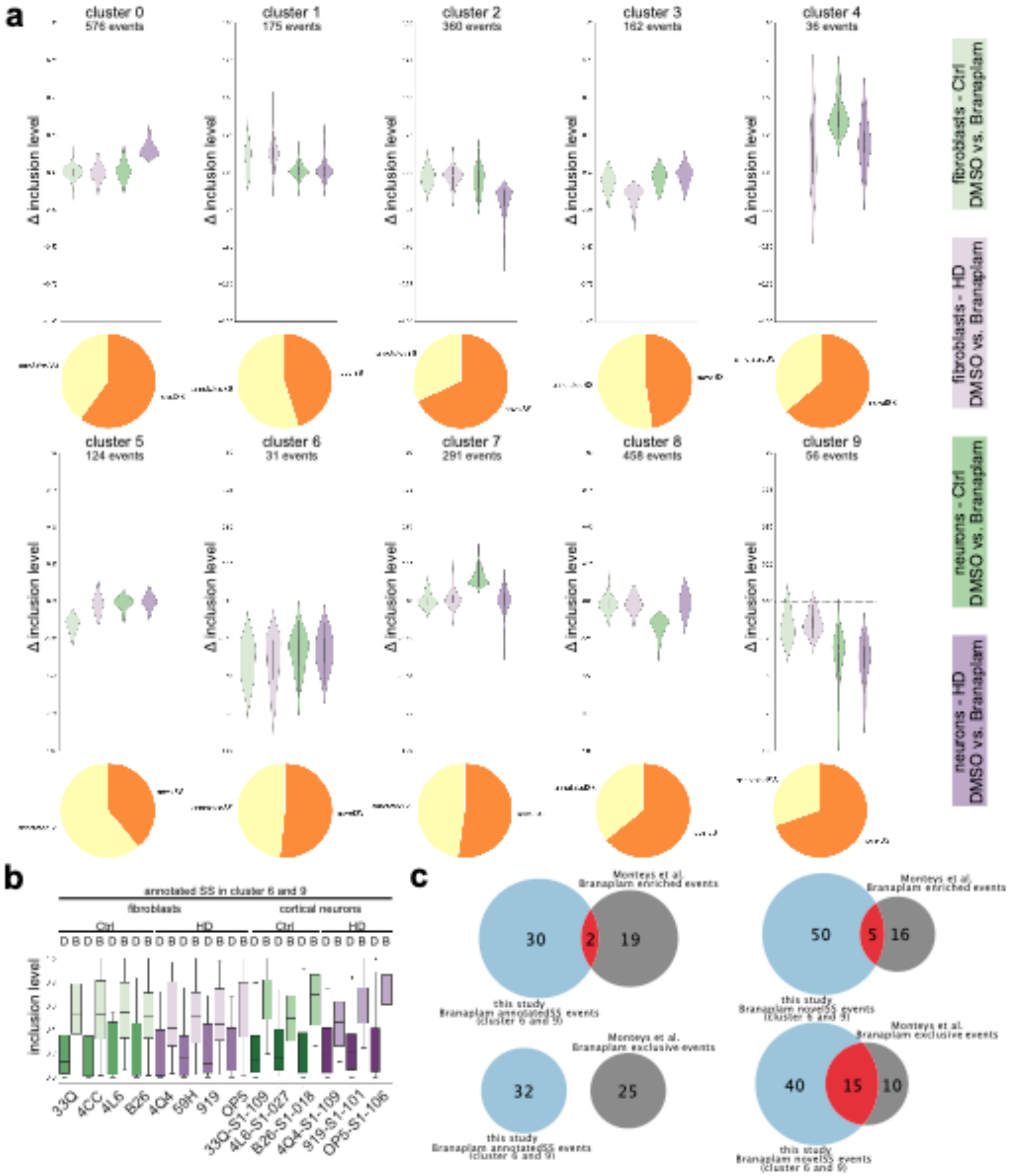

Supplementary Figure 5 Depiction of inclusion level differences of cluster 0 - 9 upon Branaplam **a** Violin plot and pie chart of alternative splicing events in cluster 0 - 10. Violin plot shows inclusion level difference in the four comparisons. Pie chart illustrates distribution of annotated (yellow) vs. non-annotated exons (novelSS, orange)

b Box plot showing inclusion values of alternative splicing events in cluster 6 and 9 that are annotated in individual samples. D: DMSO, B: Branaplam.

c Venn diagrams depicting overlaps (red) of Branaplam annotatedSS or novelSS events (from cluster 6 and 9) (light blue) from this study with events that are found to be enriched or exclusive upon Branaplam treatment in HEK293 cells from Monteys et al. (gray) [15].

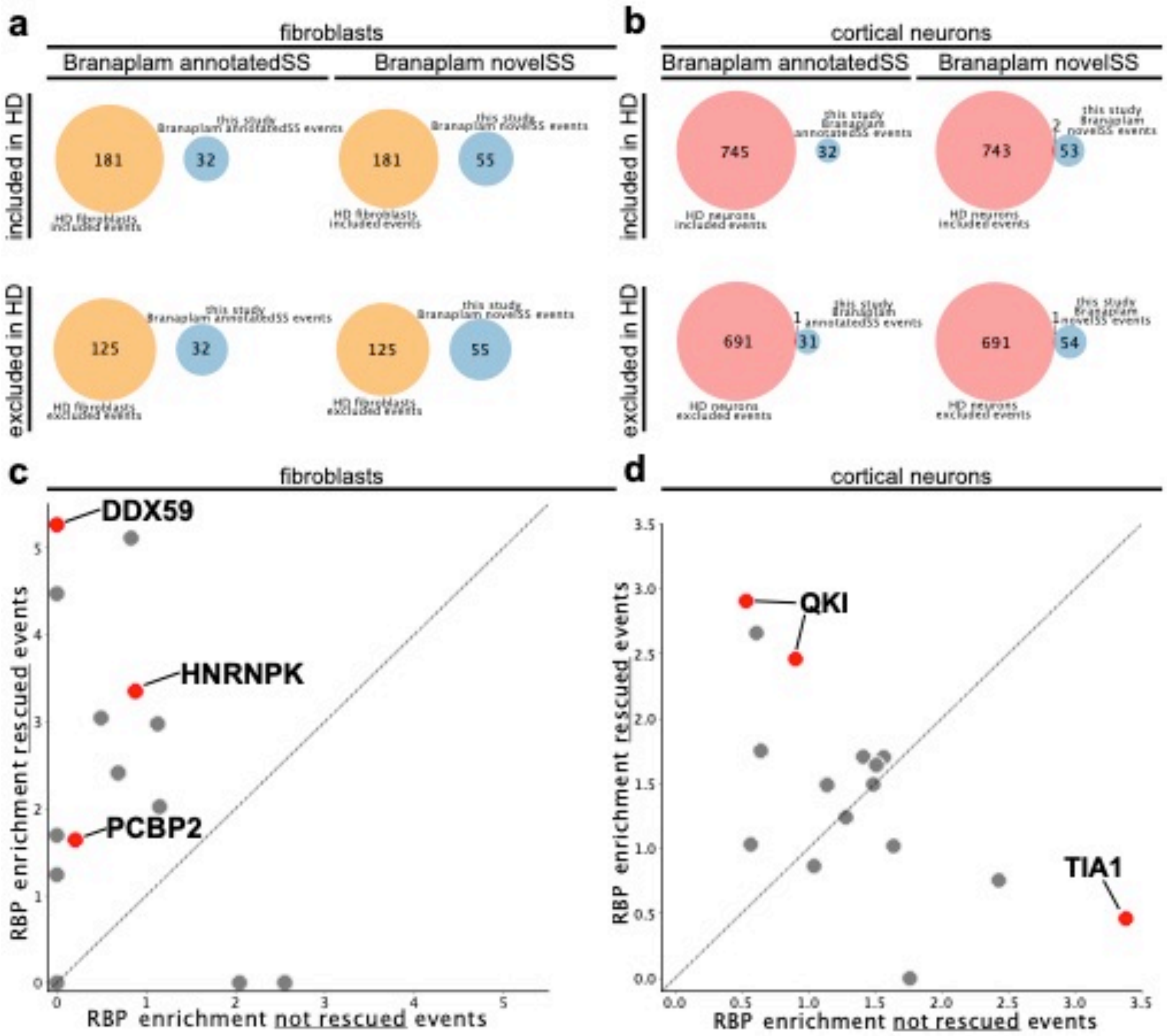

Supplementary Figure 6 Investigation of direct effects of Branaplam on rescue of aberrant alternative splicing and RBP binding enriched in rescued events

a Venn diagrams depicting overlaps (red) of Branaplam annotatedSS or novelSS events (from cluster 6 and 9) (light blue) from this study with HD alternative splicing events included or excluded in fibroblasts (orange).

b Venn diagrams depicting overlaps (red) of Branaplam annotatedSS or novelSS events (from cluster 6 and 9) (light blue) from this study with HD alternative splicing events included or excluded in cortical neurons (light red).

c Scatter plot depicting RBP enrichment in HD fibroblast AS events recued (y) and not rescued (x) by Branaplam. RBP enrichment over background was calculated for RBPs where binding was enriched in all HD fibroblast AS events (Figure 2G). In red are RBPs with significantly different

enrichment in binding in rescued vs. non rescued events (determined by Fisher's exact test, $p<0.05$).

d Scatter plot depicting RBP enrichment in HD cortical neuron AS events recued (y) and not rescued (x) by Branaplam. RBP enrichment over background was calculated for RBPs where binding was enriched in all HD cortical neuron AS events (Figure 2H). In red are RBPs with significantly different enrichment in binding in rescued vs. non rescued events (determined by Fisher's exact test, $p<0.05$).
