# Supplementary material for: An alternative splicing modulator decreases mutant HTT and improves the molecular fingerprint in Huntington’s disease patient neurons
Source: Nat Commun. 2022 Nov 10;13:6797. doi: 10.1038/s41467-022-34419-x (PMC9649613; doi:10.1038/s41467-022-34419-x)
Supplement: Supplementary file 3 — Reporting Summary [file 41467_2022_34419_MOESM3_ESM.pdf]

Corresponding author(s): Beate Winner
Juergen Winkler

Last updated by author(s): Oct 1, 2022

Reporting Summary

Nature Portfolio wishes to improve the reproducibility of the work that we publish. This form provides structure for consistency and transparency in reporting. For further information on Nature Portfolio policies, see our Editorial Policies and the Editorial Policy Checklist.

Statistics

For all statistical analyses, confirm that the following items are present in the figure legend, table legend, main text, or Methods section.

n/a	Confirmed	
☐	☒	The exact sample size (*n*) for each experimental group/condition, given as a discrete number and unit of measurement
☐	☒	A statement on whether measurements were taken from distinct samples or whether the same sample was measured repeatedly
☐	☒	The statistical test(s) used AND whether they are one- or two-sided<br>*Only common tests should be described solely by name; describe more complex techniques in the Methods section.*
☐	☒	A description of all covariates tested
☐	☒	A description of any assumptions or corrections, such as tests of normality and adjustment for multiple comparisons
☐	☒	A full description of the statistical parameters including central tendency (e.g. means) or other basic estimates (e.g. regression coefficient) AND variation (e.g. standard deviation) or associated estimates of uncertainty (e.g. confidence intervals)
☐	☒	For null hypothesis testing, the test statistic (e.g. F, t, r) with confidence intervals, effect sizes, degrees of freedom and P value noted<br>*Give P values as exact values whenever suitable.*
☐	☒	For Bayesian analysis, information on the choice of priors and Markov chain Monte Carlo settings
☐	☒	For hierarchical and complex designs, identification of the appropriate level for tests and full reporting of outcomes
☒	☐	Estimates of effect sizes (e.g. Cohen's *d*, Pearson's *r*), indicating how they were calculated

Our web collection on statistics for biologists contains articles on many of the points above.

Software and code

Policy information about availability of computer code

Data collection

flow cytometry data collection: CytExpert, Beckman Coulter, https://www.beckman.com/flow-cytometry/instruments/cytoflex/software

Data analysis

Trimmomatic (version 0.39), Bolger, et al., 2014, http://www.usadellab.org/cms/?page=trimmomatic;
STAR (version 2.7.9a), Dobin et al., 2013 https://github.com/alexdobin/STAR;
Samtools (version 1.9.0), Li et al, 2009, http://samtools.sourceforge.net/;
Python 3.7,1, Python Software Foundation, https://www.python.org/;
GraphPad Prism 9, GraphPad Software, Inc. http://graphpad.com/scientific-software/prism;
Pybedtools (version 0.8.0), Dale et al., 2011, https://daler.github.io/pybedtools/;
Subread (version 2.8.2), Liao et al., 2014, http://subread.sourceforge.net/;
ZEN blue (version 2.3), Zeiss, https://www.zeiss.com/microscopy/us/products/microscope-software.html;
rMATS (version 4.2.0), Shen et al., 2014, http://rnaseq-mats.sourceforge.net/;
Fiji (version 2.1.0), Schindelin et al, 2012, https://imagej.net/Fiji;
CytExpert (version 2.4), Beckman Coulter, https://www.beckman.com/flow-cytometry/instruments/cytoflex/software;
Scikit-learn (version 0.20.1), Pedregosa et al., 2011, https://scikit-learn.org/;
kvector (version 0.1.0), https://github.com/olgabot/kvector

For manuscripts utilizing custom algorithms or software that are central to the research but not yet described in published literature, software must be made available to editors and reviewers. We strongly encourage code deposition in a community repository (e.g. GitHub). See the Nature Portfolio guidelines for submitting code & software for further information.

Data

Policy information about availability of data

All manuscripts must include a data availability statement. This statement should provide the following information, where applicable:

- Accession codes, unique identifiers, or web links for publicly available datasets
- A description of any restrictions on data availability
- For clinical datasets or third party data, please ensure that the statement adheres to our policy

All intermediate files (e.g. rMATS outputs, counts, quantifications) are submitted within the source data. Due to the European General Data Protection Regulation and specifically patient consent of study participants who donated biological material used for the generation of the RNA-seq datasets, access will be granted via the European Genome Phenome Archive (EGA, ega-archive.org). The fibroblast RNA-seq dataset is available under the accession number EGAD00001008807 and the cortical neuron RNA-seq dataset is available under the accession number EGAD00001008808. Furthermore, any additional data and information may be acquired from the corresponding author upon reasonable request.

Field-specific reporting

Please select the one below that is the best fit for your research. If you are not sure, read the appropriate sections before making your selection.

☒ Life sciences ☐ Behavioural & social sciences ☐ Ecological, evolutionary & environmental sciences

For a reference copy of the document with all sections, see nature.com/documents/nr-reporting-summary-flat.pdf

Life sciences study design

All studies must disclose on these points even when the disclosure is negative.

Sample size	No statistical methods were used to determine the sample size in this study. The individual sample size is clearly indicated for each experiment. A sample size of >=3 in each group was chosen to allow estimation of error and accuracy of values and statistical analysis. We included each 1 fibroblast/iPSC lines from 4 HD patients and 4 control, making a total of 8 individuals included in this study for all experiments but the RNA-seq in cortical neurons that was performed with n=3 HD and n=3 Ctrl lines.
Data exclusions	no data were excluded
Replication	All data are gathered from one parallel culture / differentiation round of all available cell lines. The individual fibroblast lines / iPSC clones used for each experiment represent our replicates.
Randomization	For the in vitro studies, we did not perform any randomization since all patient and control lines were investigated for treated and untreated conditions. To reduce variability, all data points depicted in a graph resulted from an experiment where all lines were processed in parallel, including cell culture, reagent batch, sample preparation and analysis.
Blinding	Blinding was not performed for in vitro studies since the investigator was aware of the disease status of each of the lines used.

Reporting for specific materials, systems and methods

We require information from authors about some types of materials, experimental systems and methods used in many studies. Here, indicate whether each material, system or method listed is relevant to your study. If you are not sure if a list item applies to your research, read the appropriate section before selecting a response.

Materials & experimental systems

n/a	Involved in the study	
☐	☒	Antibodies
☐	☒	Eukaryotic cell lines
☒	☐	Palaeontology and archaeology
☒	☐	Animals and other organisms
☐	☒	Human research participants
☒	☐	Clinical data
☒	☐	Dual use research of concern

Methods

n/a	Involved in the study	
☒	☐	ChIP-seq
☐	☒	Flow cytometry
☒	☐	MRI-based neuroimaging

Antibodies

Antibodies used	mouse, 2B7, Coriell, #CH03023, 25.ug/ml<br>mouse, MW1, Sigma-Aldrich, #MABN2427, 5ug/ml

rabbit. D7F7, Cell Signaling, #5656, 0.5ug/ml
rat anti CTIP2, Abcam, ab18465, 1:500
mouse anti beta-III-Tubulin, Promega, G7121, 1:1000
rabbit anti PAX6, Biolegend, 901301, 1:200
mouse anti Nestin, Millipore, MAB5326, 1:500
rabbit RBFOX2: A300-864A, Bethyl Laboratories Inc., 1:1000
rabbit ILF3: A303-651A-T, Bethyl Laboratories Inc., 1:2000
rabbit QKI: A300-183A-T, Bethyl Laboratories Inc., 1:2000
rabbit U2AF2: A303-667A-T, Bethyl Laboratories Inc., 1:2000
rabbit TIAL1: RN059PW, MBL international, 1:1000
anti-PAX6-APC, Miltenyi Biotech, 130-123-267, 1:100
anti-NESTIN-PerCp-Cy5.5, BD Bioscience, 561231, 1:100
anti-bIII-Tubulin-AF405, NovusBio, NB600-1018AF405, 1:100
anti-CTIP2-FITC, Abcam, ab123449, 1:100
anti-SOX2-PerCp-Cy5.5, BD Bioscience, 561506, 1:100

Validation

All antibodies were validated by vendors. Additionally, all FACS antibodies for neuronal markers were also applied to iPSC that do not express the respective marker (negative control).
MSD: 2B7, Coriell, #CH03023, https://www.coriell.org/0/sections/Search/Sample_Detail.aspx?Ref=CH03023&PgId=166
MSD MW1, Sigma-Aldrich, #MABN2427, 5ug/ml, WB with and witout polyQ repeats, https://www.sigmaaldrich.com/DE/de/product/mm/mabn2427
MSD D7F7, Cell Signaling, #5656, 0.5ug/ml, WB rat and mouse brain tissue, ICH mouse and rat brain tissue, IHF rat hippocampus, https://www.cellsignal.de/products/primary-antibodies/huntingtin-d7f7-xp-rabbit-mab/5656
IF: anti CTIP2, Abcam, ab18465, 1:500, WB Jurkat cells and mouse brain tissue, ICC/ICF neonatal mouse hippocampal neurons, FC Jurkat cells, https://www.abcam.com/ctip2-antibody-25b6-ab18465.html
IF: mouse anti beta-III-Tubulin, Promega, G7121, 1:1000, The antibody has been tested to perform in frozen and paraffin-embedded sections of rat brain, cerebellum and spinal cord, human and rat fetal CNS progenitor cell cultures and adult human paraffin-embedded brain, https://www.promega.de/products/protein-detection/primary-and-secondary-antibodies/anti_betaiii-tubulin-mab/?catNum=G7121
IF: rabbit anti PAX6, Biolegend, 901301, 1:200, Each lot of this antibody is quality control tested by Western blotting and formalin-fixed paraffin-embedded immunohistochemical staining of brain tissue, https://www.biolegend.com/fr-fr/products/purified-anti-pax-6-antibody-11511
IF: mouse anti Nestin, Millipore, MAB5326, 1:500, Evaluated by western blot on Huvec cell lysate, https://www.merckmillipore.com/DE/de/product/Anti-Nestin-Antibody-clone-10C2,MM_NF-MAB5326
WB: rabbit RBFOX2: A300-864A, Bethyl Laboratories Inc., 1:1000, WB HEK293T cells/ 3T3, TCMK-1, CT26 and C6 cells/ U2OS, HEK293T, Hep-G2, HeLa and GaMG cells, ICH of FFPE section of human lung carcinoma, https://www.fortislife.com/products/primary-antibodies/rabbit-anti-rbm9-antibody/BETHYL-A300-864
WB: rabbit ILF3: A303-651A-T, Bethyl Laboratories Inc., 1:2000, WB HeLa, IP HEK293T and jurkat cells, ICH FFPE section of human breast carcinoma, https://www.fortislife.com/products/primary-antibodies/rabbit-anti-nf90-antibody/BETHYL-A303-651
WB: rabbit QKI: A300-183A-T, Bethyl Laboratories Inc., 1:2000, WB HeLa, HEK293T and mouse NIH, IP HeLa, ICH FFPE sections of human testicular seminoma, and mouse renal carcinoma, https://www.fortislife.com/products/primary-antibodies/rabbit-anti-qki-antibody/BETHYL-A300-183
WB: rabbit U2AF2: A303-667A-T, Bethyl Laboratories Inc., 1:2000, WB HeLa, Hek293T and Jurkat cells, ICH of human breast carcionma, https://www.fortislife.com/products/primary-antibodies/rabbit-anti-u2af65-antibody/BETHYL-A303-666
WB: rabbit TIAL1: RN059PW, MBL international, 1:1000, Western blot analysis of TIAL1 in 293T (1), HeLa (2), K562 (3), Rat1 (4) and CHO (5) using RN059PW. Immunoprecipitation of TIAL1 from HeLa with normal rabbit IgG (1) or RN059PW (2), https://www.mblintl.com/products/rn059pw/
FACS: anti-PAX6-APC, Miltenyi Biotech, 130-123-267, 1:100, own validation by lack of expression in iPSC and vendor: https://www.miltenyibiotec.com/DE-en/products/pax-6-antibody-anti-human-reafinity-rea507.html?countryRedirected=1#apc:30-tests-in-60-ul
FACS: anti-NESTIN-PerCp-Cy5.5, BD Bioscience, 561231, 1:100, own validation by lack of expression in iPSC and vendor: FC of Nestin in rat glioma/ C6 cells and inhuman Neural Stem cellsderived from H9 human embryonic stem cells, https://www.bdbiosciences.com/en-nz/products/reagents/flow-cytometry-reagents/research-reagents/single-color-antibodies-ruo/percp-cy-5-5-mouse-anti-nestin.561231
FACS: anti-bIII-Tubulin-AF405, NovusBio, NB600-1018AF405, 1:100, own validation by lack of expression in iPSC and vendor: https://www.novusbio.com/products/beta-iii-tubulin-antibody-tu-20_nb600-1018af405
FACS: anti-CTIP2-FITC, Abcam, ab123449, 1:100, own validation by lack of expression in iPSC and vendor: ICC/ICF in Jurkat cells, FC in Jurkat cells and SH-SY5Y cells, https://www.abcam.com/fitc-ctip2-antibody-25b6-ab123449.html
FACS: -SOX2-PerCp-Cy5.5, BD Bioscience, 561506, 1:100, vendor, FC of Sox2 on human embryonic stem cells (H9), ICC/ICF on human ES(H9)-derived neural stem cells, https://www.bdbiosciences.com/en-au/products/reagents/flow-cytometry-reagents/research-reagents/single-color-antibodies-ruo/percp-cy-5-5-mouse-anti-sox2.561506

Eukaryotic cell lines

Policy information about cell lines

Cell line source(s)

All used cell lines were generated at the University Hosptial Erlangen: UKERf4Q4, UKERfOP5, UKERf59H, UKERf919, UKERf33Q, UKERfB26, UKERf4CC, UKERf4L6, UKERi4Q4-S1-105, UKERi4Q4-S1-109, UKERiOP5-S1-106, UKERiOP5-S1-108, UKERi59H-S1-101, UKERi59H-S1-103, UKERi59H-S1-108, UKERi919-S1-101, UKERi33Q-S1-109, UKERiB26-S1-007, UKERiB26-S1-018, UKERi4CC-S1-007, UKERi4CC-S1-015, UKERi4L6-S1-027, UKERi4L6-S1-032

Authentication

None of the cell lines were authenticated

Mycoplasma contamination	Cells were tested bi-weekly for mycoplasma, all cells tested negative
Commonly misidentified lines (See ICLAC register)	No commonly misidentified lines were used.

Human research participants

Policy information about studies involving human research participants

Population characteristics	UKERf4Q4, female, 44 years old, 16 and 40 CAG repeats, diagnosis Huntington's disease<br>UKERfOP5, male, 54 years old, 15 and 39 CAG repeats, diagnosis Huntington's disease<br>UKERf59H, male, 26 years old, 18 and 50 CAG repeats, diagnosis Huntington's disease<br>UKERf919, female, 23 years old, 28 and 57 CAG repeats, diagnosis Huntington's disease<br>UKERf33Q, female, 45 years old, 16 and 16 CAG repeats, diagnosis healthy control<br>UKERfB26, male, 43 years old, 17 and 21 CAG repeats, diagnosis healthy control<br>UKERf4CC, male, 52 years old, 18 and 19 CAG repeats, diagnosis healthy control<br>UKERf4L6, male, 32 years old, 16 and 17 CAG repeats, diagnosis healthy control
Recruitment	HD Patients and controls were recruited through the local movement disorders outpatient clinic at the University Hospital Erlangen, Germany. HD patients were included if they fit the clinical criteria for HD and diagnosis was confirmed on a molecular genetic level. Controls were recruited to get a sex and age matched cohort that has no history of neurodegenerative disorders
Ethics oversight	Ethics oversight and approval was done by the Ethikkommission der Friedrich-Alexander Universitaet Erlangen-Nuernberg, https://www.ethikkommission.fau.de/, Nr. 4120 and 259_17B: Generierung von humanen neuronalen Modellen bei neurodegenerativen Erkrankungen.

Note that full information on the approval of the study protocol must also be provided in the manuscript.

Flow Cytometry

Plots

Confirm that:

☒ The axis labels state the marker and fluorochrome used (e.g. CD4-FITC).

☒ The axis scales are clearly visible. Include numbers along axes only for bottom left plot of group (a 'group' is an analysis of identical markers).

☒ All plots are contour plots with outliers or pseudocolor plots.

☒ A numerical value for number of cells or percentage (with statistics) is provided.

Methodology

Sample preparation	For flow cytometry, cells were dissociated using Accutase for 30 mins at 37°C and resuspended in FC buffer (2% FCS, 0.01% sodium azide in PBS). Cells were dispensed into 5 ml tubes (Sarstedt) at 500,000 cells per well. For intracellular antigens, cells were fixed and permeabilized using 100ul BD Fixation/Permeabilization Solution (BD Bioscience) for 10 mins, then 1ml of BD Perm/Wash Buffer was added, cells were incubated for 5 mins and subsequently centrifuged at 1,500 rpm for 3 mins. For intracellular staining of cortical progenitors anti-PAX6-APC (130-123-267, Miltenyi Biotech, 1:100) and anti-NESTIN-PerCp-Cy5.5 (561231, BD Bioscience, 1:100) for an additional 30 mins. After a wash step, cells were resuspended in 350µl FACS buffer containing DAPI (1µg/ml). For intracellular staining of neurons, cells were stained using anti-bIII-Tubulin-AF405 (NB600-1018AF405, NovusBio, 1:100) or anti-CTIP2-FITC (ab123449, Abcam, 1:100) for 30 mins. Additional controls included applying an antibody solution without one antibody in the full cocktail (“minus 1 control”) that was used to determine potential bleed-through of the fluorophores.
Instrument	Cytoflex S (laser 405nm, 488nm, 561nm, 638nm; Beckman Coulter)
Software	CytExpert 2.4
Cell population abundance	No cells were sorted, only FACS analysis was applied. The abundance of the individual populations are shown in the respective figures.
Gating strategy	Debris was excluded by plotting FSC-A and FSC-Width. Potential singlets were determined in the FSC-A and FSC-H plot. Cells to be analyzed were gated in the FSC-A and SSC-A plot. Gates for antibody- fluorophores for neural/neuronal antigens were determined by using stained, undifferentiated iPSC as a negative control. For the Casp3/7 stain, unstained and MNs treated with sodium arsenite were used to determine the gate.

☒ Tick this box to confirm that a figure exemplifying the gating strategy is provided in the Supplementary Information.
