# Supplementary figures and images for: An alternative splicing modulator decreases mutant HTT and improves the molecular fingerprint in Huntington’s disease patient neurons
Source: Nat Commun. 2022 Nov 10;13:6797. doi: 10.1038/s41467-022-34419-x (PMC9649613; doi:10.1038/s41467-022-34419-x)

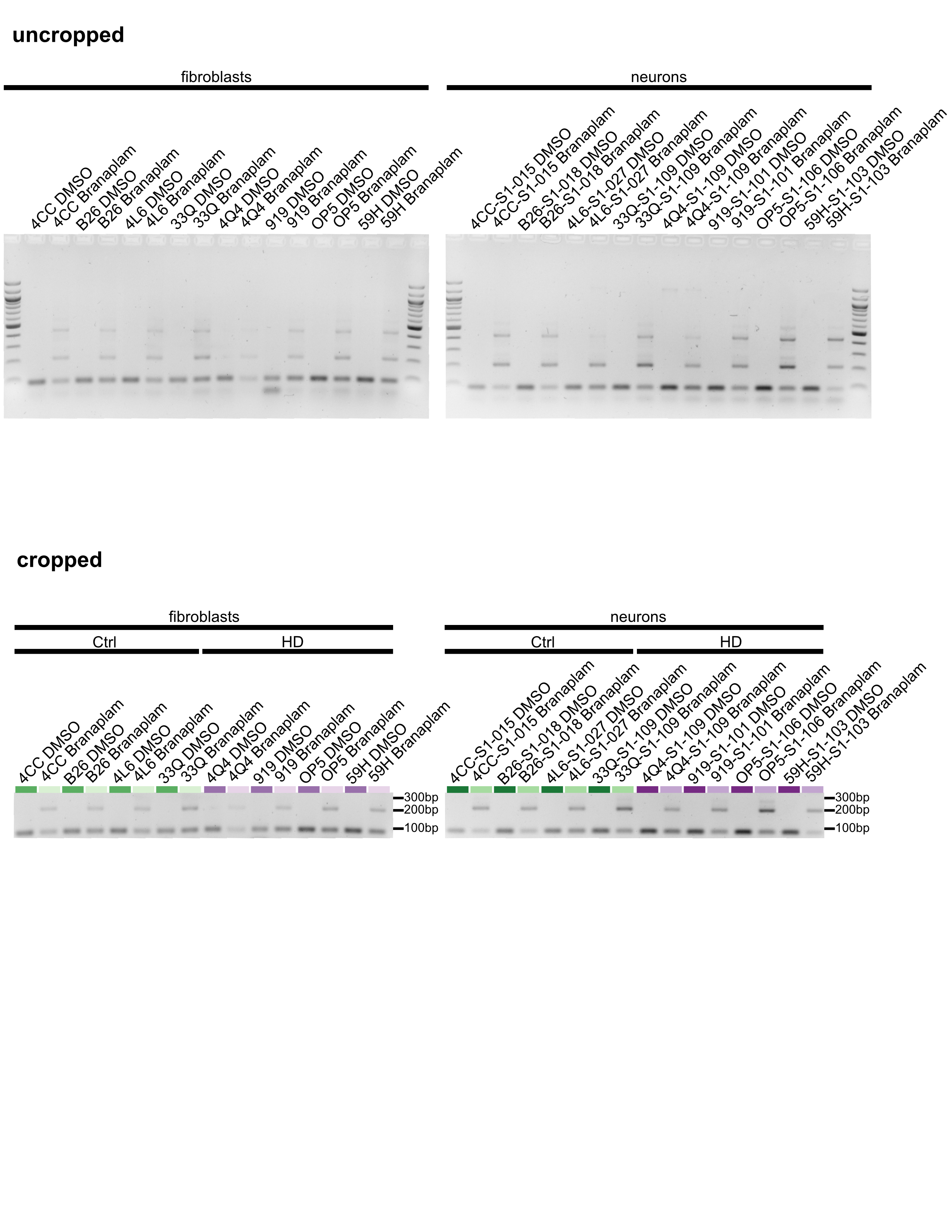

Supplement: Supplementary file 4 — Source Data [file 41467_2022_34419_MOESM4_ESM.zip › Source_Data/Figure-5d-and-f_HTT_novel_exon.tiff]

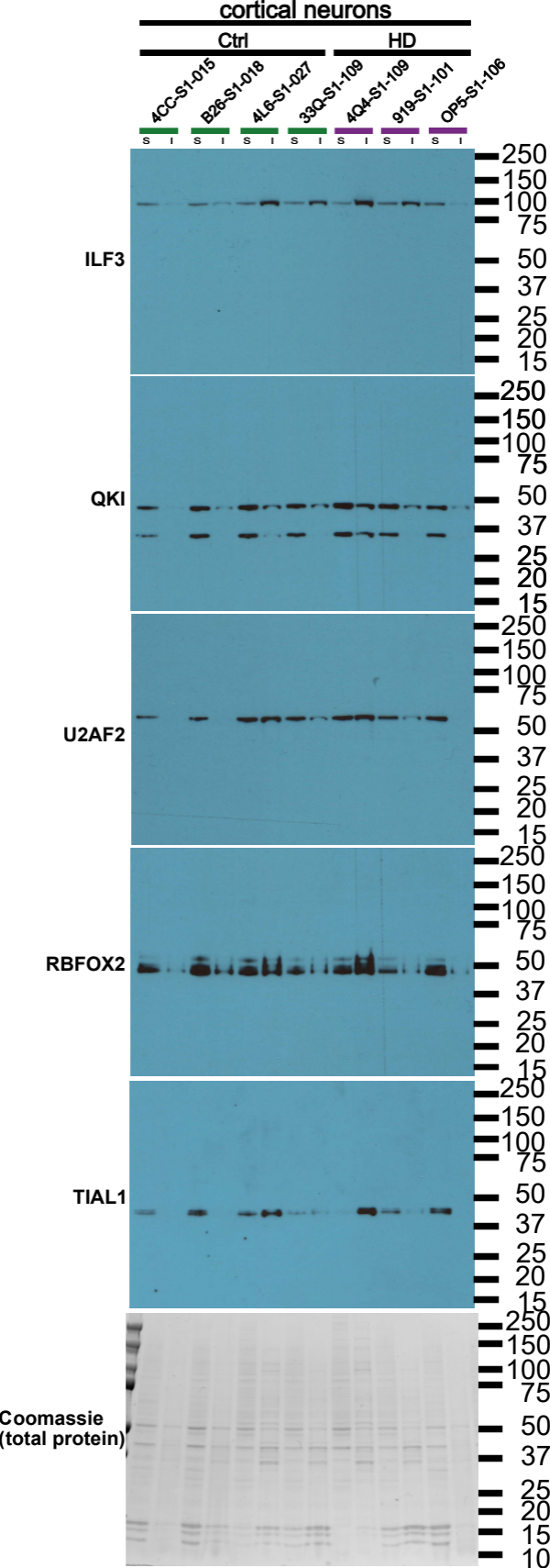

Supplement: Supplementary file 4 — Source Data [file 41467_2022_34419_MOESM4_ESM.zip › Source_Data/Supplementary-Figure-3a_whole_blots_sol-insol_HD_cortical_d50_Coomassie.pdf]
